# Supplementary material for: Collective navigation can facilitate passage through human-made barriers by homeward migrating Pacific salmon
Source: Proc Biol Sci. 2020 Oct 21;287(1937):20202137. doi: 10.1098/rspb.2020.2137 (PMC7661290; doi:10.1098/rspb.2020.2137)
Supplement: Electronic Supplementary Material [file rspb20202137supp1.pdf]

Electronic Supplementary Material for: Collective navigation can facilitate passage through human-made barriers by homeward migrating Pacific salmon

Connie Okasaki, Matthew L. Keefer,
Peter A. H. Westley & Andrew M. Berdahl

September 28, 2020

1 Statistical Analysis

We fit two proportional hazards (PH) models and one logistic model: these models are termed "finding," "ladder," and "committing" respectively. Each model was fit for Chinook and sockeye separately, since we expected inter-species differences in most or all coefficients. The PH models were fit in `R` [7] using the `phreg` function from the `eha` package [1]. All models included daily counts of conspecifics. Since counts are available for each ladder, we used ladder-specific counts for the "ladder" and "commit" processes; however since a fish might approach either ladder upon entering the tailrace we used dam-level counts for the "finding" process.

All models controlled for temperature [3], dam identity, and a binary diel effect [4]. For the finding and committing processes we included a spill effect, but since fish cannot detect spill from within the fishway, no spill effect was included in the ladder models.

For all models we included a binary covariate (`middle`) indicating whether a fish's tailrace entry was in the middle (2nd and 3rd) quartiles. This variable controlled for the effect of run timing on fish condition and passage speed, since timing is highly correlated with density (very high densities near the modal date) and might otherwise confound our analysis.

For Chinook we included a binary variable (`spring`) indicating whether a fish was labeled spring- or summer-run. For sockeye we included a three-level factor variable indicating whether a fish was sourced to the Wenatchee, the Okanagan, or to any other site. The Wenatchee and Okanogan tributaries each accounted for about a third of the sockeye in our sample.

Two of our predictors — `temperature` and `day` — had strong prior evidence from the literature [3, 4]. To reduce the number of variables being selected and the risks associated with model dredging, we included these variables a priori. The remainder of the predictors we selected using `AICc`. For consistency, however, we wished to fit structurally identical models to each set of species/process submodels. We examined the `AICc` for each set of models and

subjectively chose what seemed to be the best-fitting overall model. Due to the extremely long tails of the count predictor, we then considered adding either an un-transformed or a log-transformed count variable to the best-fitting null model. In most cases there was no discernible difference in `AICc` between the two, but in several cases the un-transformed variable was selected. For consistency, despite the long tails, we used the un-transformed version to conduct the remainder of our analysis.

The classical form of the PH model uses a partial likelihood function and does not specify the baseline hazard function, but in order to simulate from our bootstrapped distributions we assumed a constant baseline hazard $\lambda_0(t) = \lambda_0$. This was visually consistent with the empirical hazard functions. However, since there is a small but non-trivial minimum time to complete each task, the empirical hazard functions are zero for a short period of time. To account for this discrepancy, we truncated each fish's timeline by a constant amount (per model) such that the minimum passage time was less than 1 minute, effectively assuming a piecewise constant baseline hazard function with the first section fixed at zero hazard. In several models there were outlier individuals detected as passing through the fishway in as little as 2 minutes. Since such passage times are implausible (fast passage times are ≈ 1 hour), we attribute this to data error and remove all records which show passage in <10 minutes. This approach conservatively includes several extraordinarily fast passage times of ≈ 30 minutes.

For each model, we dropped any fish with censored measurements. Censoring is a standard concern for time-to-event models, and occurs when an event is not observed to occur because an individual leaves observation. This could occur when a fish passed but was not detected by a telemetry antenna, or because a fish died in a tailrace. Incorrect assumptions regarding censoring are known to bias inference in PH models [5]. We did not account for censoring because, for each system, we used essentially only two measurements for each fish: "entry" and "exit." A censoring analysis would require follow-up measurements between entry and exit to verify that an individual is still being detected but has not yet exited. Although the aerial tailrace antenna misses a large number of measurements, the underwater fishway antennas miss far fewer. Only a very small portion ($\approx 1\%$) of fish are observed to enter but not exit the fishway. Since a very similar portion of fish are observed to exit but not enter, we attribute this censoring almost entirely to randomly missed telemetry detections rather than mortality. Since individuals were therefore dropped from the dataset independently of hazard we lose little information and incur very little bias in this manner.

Finally, we conducted a parametric bootstrap test on the count coefficient to detect density effects, using our best-fitting null models. Since these models do not contain reverse causality, they provide valid, unbiased fits under the null hypothesis. To each simulation, we fit a model with an effect of count. Assuming our null model is correctly specified, comparing our fitted count coefficients to these null distributions provide valid p-values to test for density effects. We do not Bonferroni correct these p-values because by the nature of our split models a single false-positive result is not statistically convincing, as we note when discussing our two marginally significant results. Instead we demand consistency between matching models, and indeed we found that several models were consistently highly significant (and would remain so even with a Bonferroni correction).

To ensure that our null models were well-specified, we conducted standard model diagnostics. For our PH models we tested the proportional hazards assumption using the

`cox.zph` function in the `survival` package [8], applied to the partial-likelihood model fits. Since the `cox.zph` function tests the proportional hazards assumption for each covariate as well as globally for each model we made 36 tests. Using a Bonferroni correction, this test identified strong differences (corrected $p < 10^{-5}$) in baseline hazard functions between dams, likely caused by differences in the layout of the monitoring arrays. We thus split our models by dam. Retesting these sub-models, we made an additional 59 tests. These tests caused us to split our Chinook ladder models by run ($p < 10^{-5}$) and to split our `sockeye.ladder.TD` models by site ($p < 0.005$; East (TDE) vs. North (TDN)). Since all of these were individual-level variables, we could not assign interactions with time, since this could (and did) result in predictions of effectively infinite survival time. We also added interaction effects between temperature and time ($p < 0.001$) in all of the Chinook ladder models. Finally we made an additional 41 tests after conducting this second level of model-splitting, with no significant results. After splitting, the Chinook ladder models each contained between 330 and 440 fish. All other models contained over 500 fish, with the exception of `sockeye.ladder.TDN` which had only 59. Our bootstrap simulations frequently produced ill-conditioned fits for this model, and we subsequently removed it from our analysis. A diagram of our analysis can be found in Figure 1.

For the `commit` models we fit one logistic regression model per species, without splitting by dam. We plotted binned residuals from the null model, which motivated the addition of a threshold variable (count $\leq$150) to the Chinook model, due to substantially higher-than-expected commit rates for these low-density fish. Of these 89 individuals, 88 of them were detected at The Dalles's east ladder. Elevated commit probability was not observed at TDE at higher densities.

Although we hypothesized positive density effects, we conducted two-sided tests in all cases to allow for the possibility of negative effects (e.g. overcrowding in the fishway).

Because of the reverse causality in our system, the null modeling approach we took was the most reliable way we knew to detect a density effect. However, this approach provides only p-values, and not confidence intervals for the true magnitude. In order to quantify magnitude, we conducted another parametric bootstrap to produce 95% confidence intervals for the count coefficient. To produce these intervals we sampled from the full model (i.e. the model with a count coefficient) and fit to these simulations new count coefficients. We calculate the confidence intervals using the hybrid method, recommended in [2] for use when estimating regression coefficients in Cox models. In all cases, the bootstrapped distribution was symmetric and centered on the fitted coefficient. This secondary bootstrap also roughly reproduced the p-values of our primary bootstrap. These two metrics lend credibility to this bootstrap, despite possible biases that might enter due to reverse causality. Thus, we also report approximate 95% confidence intervals for the magnitude of the density coefficient, calculated from these bootstrapped distributions. All bootstraps included >2000 simulations.

To speed up calculation we did bootstrap simulations in parallel using 24 cores of a 48 core Windows compute server. We parallelized using the R package `parallel` [7] and set our random seeds using [6]'s method.

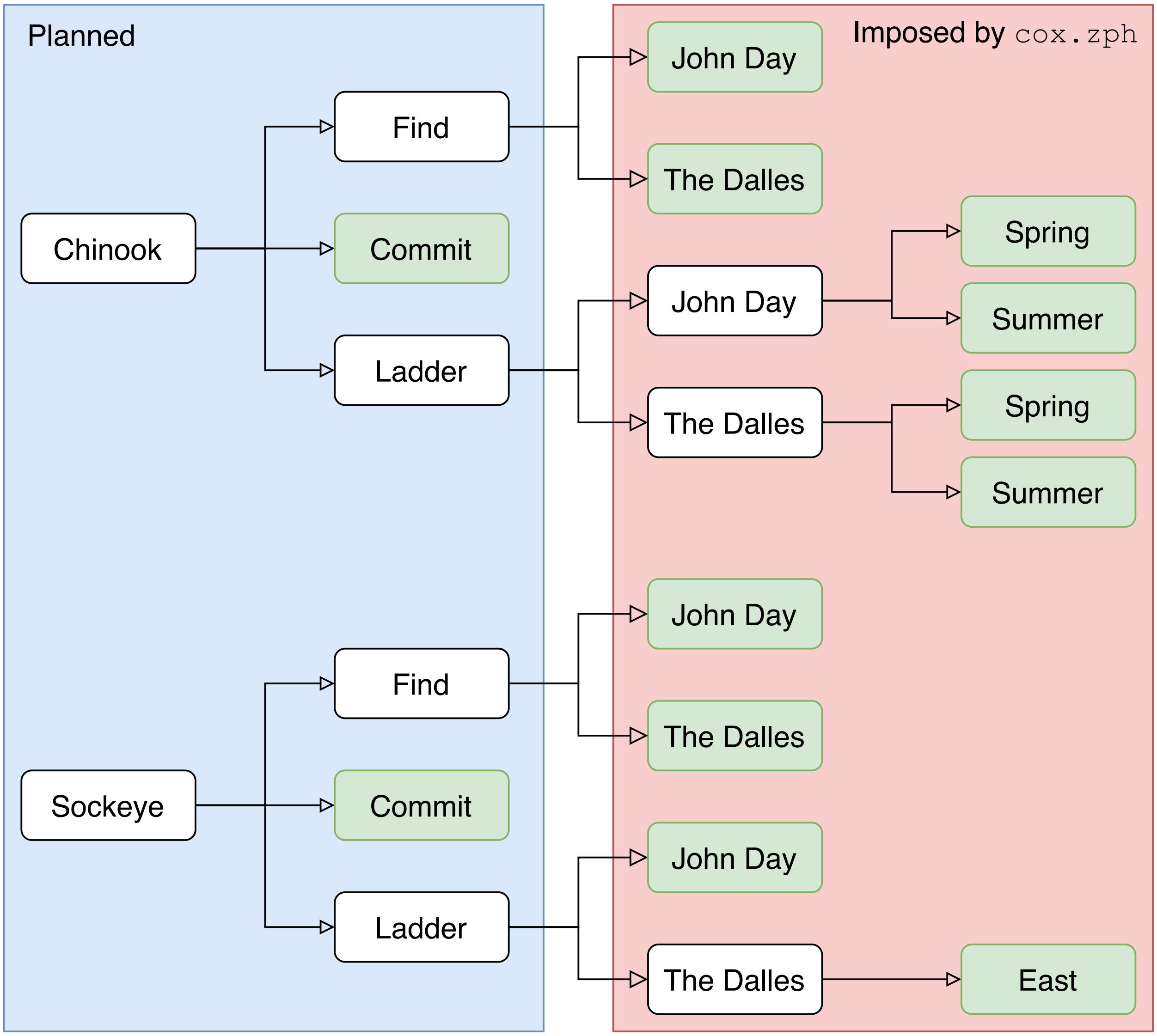

Figure 1: A diagram of the models we fit. Originally we planned to fit the 6 models shown in blue on the left. However, due to model diagnostic violations (specifically violations detected by `cox.zph`) we were forced to split further to allow baseline hazard functions to differ between factors. These more specific final models are shown in red. Our final set of models which passed all model diagnostics are shown in green.

2 Model Summaries

Below, we have included the model output for our final selected models. For the three models where a highly significant density effect was detected we include that density effect. Otherwise we include the best-fitting null model.

```
Find Chin JD
Call:
phreg(formula = Surv(tstart, tstop, success) ~ day + middle +
    spring + temp.c + spill + chin, data = find.chin.JD.trunc,
    dist = "pch")

Covariate           W.mean      Coef Exp(Coef)  se(Coef)    Wald p
day                  0.577     2.245     9.445     0.134     0.000
middle
           FALSE    0.375     0         1           (reference)
            TRUE    0.625    -0.523     0.593     0.075     0.000
spring
           FALSE    0.364     0         1           (reference)
            TRUE    0.636     0.044     1.045     0.113     0.697
temp.c              -0.297     0.616     1.852     0.120     0.000
spill                0.682     0.204     1.227     0.108     0.058
chin                -0.048     0.559     1.748     0.090     0.000


Events                     804
Total time at risk         5255.9
Max. log. likelihood       -1997.2
LR test statistic          632.58
Degrees of freedom         6
Overall p-value            0
```

```
Find Chin TD
Call:
phreg(formula = Surv(tstart, tstop, success) ~ day + middle +
    spring + temp.c + spill + chin, data = find.chin.TD.trunc,
    dist = "pch")

Covariate          W.mean      Coef Exp(Coef)  se(Coef)    Wald p
day                 0.575     2.889    17.978     0.187     0.000
middle
           FALSE    0.484     0         1           (reference)
            TRUE    0.516     0.155     1.168     0.079     0.051
spring
           FALSE    0.345     0         1           (reference)
            TRUE    0.655     0.654     1.923     0.164     0.000
temp.c             -0.322     1.299     3.667     0.191     0.000
spill               0.777    -0.075     0.928     0.191     0.695
chin                0.093     0.324     1.383     0.082     0.000


Events                     751
Total time at risk         6445.7
Max. log. likelihood       -2030.9
LR test statistic          669.09
Degrees of freedom         6
Overall p-value            0
```

```
Fishway Chin JD Spring
Call:
phreg(formula = Surv(tstart, tstop, success) ~ day + site.JDN +
    temp.c + temp.ct, data = ladder.chin.JD.spring.trunc, dist = "pch")

Covariate          W.mean      Coef Exp(Coef)  se(Coef)    Wald p
day                 0.755     2.945    19.002     0.382     0.000
site.JDN
           FALSE    0.738     0         1           (reference)
            TRUE    0.262     0.203     1.225     0.102     0.047
temp.c             -0.578     0.296     1.344     0.154     0.055
temp.ct            -2.409    -0.003     0.997     0.012     0.798


Events                     439
Total time at risk           1707
Max. log. likelihood      -935.96
LR test statistic          198.41
Degrees of freedom         4
Overall p-value            0
```

```
Fishway Chin JD Summer
Call:
phreg(formula = Surv(tstart, tstop, success) ~ day + site.JDN +
    temp.c + temp.ct, data = ladder.chin.JD.summer.trunc, dist = "pch")

Covariate          W.mean      Coef Exp(Coef)  se(Coef)    Wald p
day                 0.797     2.360    10.594     0.282     0.000
site.JDN
           FALSE    0.436     0         1           (reference)
            TRUE    0.564    -0.024     0.977     0.110     0.829
temp.c              0.292    -0.651     0.521     0.249     0.009
temp.ct             0.619     0.437     1.548     0.048     0.000


Events                    345
Total time at risk        1101.5
Max. log. likelihood      -681.38
LR test statistic         128.25
Degrees of freedom        4
Overall p-value           0
```

```
Fishway Chin TD Spring
Call:
phreg(formula = Surv(tstart, tstop, success) ~ day + site.TDN +
    temp.c + temp.ct, data = ladder.chin.TD.spring.trunc, dist = "pch")

Covariate          W.mean      Coef Exp(Coef)  se(Coef)    Wald p
day                 0.589     2.783    16.161     0.257     0.000
site.TDN
           FALSE    0.820     0         1           (reference)
            TRUE    0.180     0.108     1.114     0.130     0.405
temp.c             -0.520    -0.342     0.711     0.191     0.074
temp.ct            -2.105     0.063     1.065     0.021     0.003


Events                    415
Total time at risk        1115.4
Max. log. likelihood      -659.5
LR test statistic         331.65
Degrees of freedom        4
Overall p-value           0
```

```
Fishway Chin TD Summer
Call:
phreg(formula = Surv(tstart, tstop, success) ~ day + site.TDN +
    temp.c + temp.ct, data = ladder.chin.TD.summer.trunc, dist = "pch")

Covariate          W.mean      Coef Exp(Coef)  se(Coef)    Wald p
day                 0.784     2.101     8.172     0.310     0.000
site.TDN
           FALSE    0.671     0         1           (reference)
            TRUE    0.329    -0.224     0.799     0.127     0.076
temp.c              0.234     0.337     1.401     0.260     0.196
temp.ct             0.658     0.048     1.049     0.047     0.309


Events                    332
Total time at risk        1017.7
Max. log. likelihood        -654
LR test statistic         99.77
Degrees of freedom        4
Overall p-value           0
```

```
Find Sock JD
Call:
phreg(formula = Surv(tstart, tstop, success) ~ day + middle +
    wenatchee + okanogan + temp.c + spill, data = find.sock.JD.trunc,
    dist = "pch")

Covariate          W.mean      Coef Exp(Coef)  se(Coef)    Wald p
day                 0.459     1.951     7.034     0.111     0.000
middle
           FALSE    0.461     0         1           (reference)
            TRUE    0.539    -0.211     0.810     0.084     0.012
wenatchee           0.187     0.381     1.463     0.114     0.001
okanogan            0.456     0.195     1.215     0.094     0.039
temp.c              0.220     0.668     1.950     0.113     0.000
spill               0.546    -0.456     0.634     0.143     0.001


Events                    609
Total time at risk        1836.4
Max. log. likelihood      -1067.7
LR test statistic         426.88
Degrees of freedom        6
Overall p-value           0
```

```
Find Sock TD
Call:
phreg(formula = Surv(tstart, tstop, success) ~ day + middle +
    wenatchee + okanogan + temp.c + spill, data = find.sock.TD.trunc,
    dist = "pch")

Covariate          W.mean      Coef Exp(Coef)  se(Coef)    Wald p
day                 0.589     2.703    14.926     0.190     0.000
middle
           FALSE    0.525     0         1           (reference)
            TRUE    0.475     0.122     1.129     0.082     0.137
wenatchee           0.195     0.400     1.491     0.112     0.000
okanogan            0.432     0.131     1.140     0.094     0.164
temp.c              0.301     0.759     2.135     0.221     0.001
spill               0.677    -0.269     0.765     0.219     0.221


Events                     616
Total time at risk         3905.7
Max. log. likelihood       -1524.1
LR test statistic          459.16
Degrees of freedom         6
Overall p-value            0
```

```
Fishway Sock JD
Call:
phreg(formula = Surv(tstart, tstop, success) ~ day + site.JDN +
    temp.c, data = ladder.sock.JD.trunc, dist = "pch")

Covariate          W.mean      Coef Exp(Coef)  se(Coef)    Wald p
day                 0.786     1.533     4.634     0.182     0.000
site.JDN
           FALSE    0.676     0         1           (reference)
            TRUE    0.324     0.743     2.102     0.082     0.000
temp.c              0.300     0.214     1.239     0.179     0.231


Events                    605
Total time at risk        2347.6
Max. log. likelihood      -1320.5
LR test statistic         209.60
Degrees of freedom        3
Overall p-value           0
```

```
Fishway Sock TD East
Call:
phreg(formula = Surv(tstart, tstop, success) ~ day + temp.c,
    data = ladder.sock.TDE.trunc, dist = "pch")

Covariate          W.mean      Coef Exp(Coef)  se(Coef)    Wald p
day                 0.784     1.199     3.315     0.156     0.000
temp.c              0.219     0.872     2.391     0.156     0.000


Events                    553
Total time at risk        1871.9
Max. log. likelihood       -1169
LR test statistic         116.59
Degrees of freedom        2
Overall p-value           0
```

```
Commit Chin

Call:
glm(formula = success ~ day + site + spring + temp.c + thresh +
    chin.specific, family = binomial, data = prob.chin)

Deviance Residuals:
    Min       1Q   Median       3Q      Max
-1.7907  -1.0415  -0.6673   1.1420   2.1301

Coefficients:
              Estimate Std. Error z value Pr(>|z|)
(Intercept)   -0.03122    0.28168  -0.111  0.91176
day           -0.04247    0.23845  -0.178  0.85863
siteJDS       -1.02322    0.17067  -5.995 2.03e-09 ***
siteTDE        0.04591    0.16864   0.272  0.78544
siteTDN        0.11066    0.28167   0.393  0.69442
springTRUE    -0.31638    0.22854  -1.384  0.16625
temp.c        -1.22897    0.26553  -4.628 3.68e-06 ***
threshTRUE     1.50867    0.28215   5.347 8.94e-08 ***
chin.specific  0.75259    0.21903   3.436  0.00059 ***
---
Signif. codes:  0 ?***? 0.001 ?**? 0.01 ?*? 0.05 ?.? 0.1 ? ? 1

(Dispersion parameter for binomial family taken to be 1)

    Null deviance: 2129.1  on 1554  degrees of freedom
Residual deviance: 1898.2  on 1546  degrees of freedom
AIC: 1916.2

Number of Fisher Scoring iterations: 4
```

Commit Sock

```
Call:
glm(formula = success ~ day + site + temp.c, family = binomial,
    data = prob.sock)

Deviance Residuals:
    Min       1Q   Median       3Q      Max
-1.7249  -1.1053   0.7476   0.7485   1.3937

Coefficients:
             Estimate Std. Error z value Pr(>|z|)
(Intercept)  0.911939   0.243638   3.743 0.000182 ***
day          0.319363   0.202688   1.576 0.115110
siteJDS     -1.401290   0.183263  -7.646 2.07e-14 ***
siteTDE     -0.098865   0.180317  -0.548 0.583497
siteTDN     -0.313573   0.375813  -0.834 0.404064
temp.c      -0.006558   0.314959  -0.021 0.983387
---
Signif. codes:  0 ?***? 0.001 ?**? 0.01 ?*? 0.05 ?.? 0.1 ? ? 1

(Dispersion parameter for binomial family taken to be 1)

    Null deviance: 1564.5  on 1224  degrees of freedom
Residual deviance: 1457.1  on 1219  degrees of freedom
AIC: 1469.1

Number of Fisher Scoring iterations: 4
```

References

[1] Göran Broström and Maintainer Göran Broström. Package 'eha', 2019.

[2] Deborah Burr. A comparison of certain bootstrap confidence intervals in the cox model. *Journal of the American Statistical Association*, 89(428):1290–1302, 1994.

[3] Christopher C Caudill, Matthew L Keefer, Tami S Clabough, George P Naughton, Brian J Burke, and Christopher A Peery. Indirect effects of impoundment on migrating fish: temperature gradients in fish ladders slow dam passage by adult chinook salmon and steelhead. *PloS one*, 8(12):e85586, 2013.

[4] Matthew L Keefer, Christopher C Caudill, Christopher A Peery, and Mary L Moser. Context-dependent diel behavior of upstream-migrating anadromous fishes. *Environmental Biology of Fishes*, 96(6):691–700, 2013.

[5] David G Kleinbaum and Mitchel Klein. *Survival analysis*, volume 3. Springer, 2010.

[6] Pierre L'ecuyer, Richard Simard, E Jack Chen, and W David Kelton. An object-oriented random-number package with many long streams and substreams. *Operations research*, 50(6):1073–1075, 2002.

[7] R Core Team. *R: A Language and Environment for Statistical Computing*. R Foundation for Statistical Computing, Vienna, Austria, 2019.

[8] Terry M Therneau and Thomas Lumley. Package 'survival'. *Survival analysis Published on CRAN*, 2:3, 2014.
